# Synthetic Turf: Health DebateTakes Root

**DOI:** 10.1289/ehp.116-a116

**Published:** 2008-03

**Authors:** Luz Claudio

In Little League dugouts, community parks, professional athletic organizations, and international soccer leagues, on college campuses and neighborhood playgrounds, even in residential yards, the question being asked is “grass or plastic?” The debate is over synthetic turf, used to blanket lawns, park spaces, and athletic fields where children and adults relax and play; the questions are whether synthetic turf is safe for human and environmental health, and whether its advantages outweigh those of natural grass. Despite or perhaps because of the fact that it is too early to definitively answer those questions, the debate is fierce.

New York City, which buys the largest amount of synthetic turf of any U.S. municipality, held a hearing 13 December 20007 on the use of synthetic turf in city parks. There is a clear need for open space in the city. The 28,700 acres of land constituting some 4,000 parks are distributed unevenly throughout the city. “Many districts have no green parks, not even one,” said Helen Sears, a city council member representing the Jackson Heights neighborhood, during the hearing.

New York City Department of Parks & Recreation commissioner Adrian Benepe wants to address the need for parks and athletic fields by installing not only natural grass fields and lawns but also synthetic turf. “With quality recreational facilities—which means, in some cases, synthetic turf fields—we will be able to better confront this issue,” he says. In New York City, he points out, at least 35 synthetic turf fields are or will be a replacement for asphalt surfaces.

Others oppose the move toward synthetic turf. “Grassroots organizations have been working hard to have pesticide use reduced or banned in places where it is unnecessary,” says Tanya Murphy, a board member of Healthy Child, Healthy World, an advocacy organization. “Now we’re going from the frying pan and into the fire when replacing grass with synthetic turf.”

The debate leaves many on the fence. Orlando Gil, an assistant research scientist at New York University and soccer coach, is weighing both alternatives: “We want children to play outside, exercise, and play sports, but with pesticides and fertilizers in grass and chemicals in artificial turf, I don’t know which to choose.”

Indeed, a dearth of research on the nonoccupational human health effects of exposure to the constituents of synthetic turf hampers the ability to make that choice with any degree of confidence. On the basis of limited toxicity data, some reports have concluded the health risks are minimal. Most agree, however, that far more research is needed before the question can be definitively answered. In the 13 December 2007 issue of *Rachel’s Democracy and Health News*, William Crain of the City College of New York Psychology Department and Junfeng Zhang of the University of Medicine & Dentistry of New Jersey School of Public Health called conclusions of minimal risk “premature.”

## A Turf History

During the 1950s, the Ford Foundation studied ways to incorporate physical fitness into the lives of young people, particularly in cities where outdoor play areas were scarce. Ford joined Monsanto Industries to create an artificial surface on which children could play sports. In 1964 the first artificial playing surface was marketed under the name Chemgrass.

Meanwhile, the first domed stadium was being built in Houston, Texas. The Astrodome, with its retractable translucent plastic ceiling, let in enough sunshine to maintain a natural grass field. But after the first baseball season, it was clear there was a problem. The plastic panes produced a glare that made it difficult for players to see the ball. This problem was solved by painting the panes black—but then the grass began to die from lack of sunlight. By the beginning of the second season, the Astros were playing on dead grass and painted dirt. At this time, production of Chemgrass was limited, but what little was available was installed in the Astrodome. By the end of the 1966 season, the material had been renamed AstroTurf. The green nylon carpet was a success.

The popularity of AstroTurf grew steadily during the 1970s and 1980s, with most of its use in professional sports arenas. However, a backlash began to unfold when players started to complain about the surfacing. The English Football Association banned synthetic turf in 1988, mainly because of complaints from athletes that it was harder than grass and caused more injuries. Similar concerns were growing in the United States. A poll conducted by the National Football League Players Association in 1995 showed that more than 93% of players believed playing on artificial surfaces increased their chances of injury. This sentiment was famously expressed by baseball player Dick Allen: “If a horse won’t eat it, I don’t want to play on it.”

The movement against AstroTurf gained traction, and many ballparks were converted to natural grass during the 1990s. One example was Giants Stadium in New Jersey, which had used AstroTurf since its construction in 1976. The stadium was refitted with a system of 6,000 removable trays of natural grass. Even the new stadium in Houston, built to replace the original Astrodome, was surfaced with grass.

In this story of grass, the balance is tilting once more against the natural kind. Natural grass, under some circumstances, cannot consistently withstand the demands of sports where a lot of running is involved. Parallel to this back-and-forth controversy over which is best have come new developments in the manufacture of synthetic turf. Several companies, including the makers of the original AstroTurf, have come on the market with new playing surfaces.

FieldTurf, for example, is made of a blended polyethylene–polypropylene material woven to simulate blades of grass. The “grass” is held upright and given some cushioning by adding a layer of infill made of recycled tires, rubber particles 3 mm in diameter or smaller. This crumb rubber infill is sometimes mixed with silica sand. Many stadiums that switched to grass from AstroTurf have since switched back to FieldTurf-style synthetic turf.

Figures from the Synthetic Turf Council, a trade organization based in Atlanta, show that 10 years ago there were 7 new-generation fields installed in the United States. Today there are 3,500. Says Geoffrey Croft, president of the nonprofit New York City Parks Advocates, which promotes public funding and increased park services, “There are millions of square feet of synthetic turf already installed on fields around the country, and not one environmental impact statement has been issued.”

## Human Health Questions

Given the relatively recent development of new-generation synthetic turf, there are unanswered questions regarding its potential effects on health and the environment, with the rubber infill one of the main sources of concern. The crumbs become airborne and can be breathed in and tracked into homes on clothes and athletic gear. There are also questions about dermal and ingestional exposures, and about ecosystem effects.

For athletes, the little black rubber pellets may seem little more than a nuisance. Others express more concern, especially when it comes to children’s exposure to the infill. Patti Wood, executive director of the nonprofit Grassroots Environmental Education, argues, “This crumb rubber is a material that cannot be legally disposed of in landfills or ocean-dumped because of its toxicity. Why on earth should we let our children play on it?”

Recycled crumb rubber contains a number of chemicals that are known or suspected to cause health effects. The most common types of synthetic rubber used in tires are composed of ethylene–propylene and styrene–butadiene combined with vulcanizing agents, fillers, plasticizers, and antioxidants in different quantities, depending on the manufacturer. Tire rubber also contains polyaromatic hydrocarbons (PAHs), phthalates, and volatile organic compounds (VOCs).

According to the Rubber Manufacturers Association, only 8 states have no restrictions on placing tires in landfills. Most of these restrictions have to do with preventing pest problems and tire fires, which release toxicants such as arsenic, cadmium, lead, nickel, PAHs, and VOCs.

Some studies suggest that the same chemicals that can be released profusely during a tire fire may also be released slowly during deterioration of crumb rubber. For instance, researchers at the Norwegian Institute of Public Health presented a report at the 2006 meeting of the International Association for Sports Surface Sciences on turf-related chemicals in indoor stadiums. The report, *Artificial Turf Pitches: An Assessment of the Health Risks for Football Players*, showed that VOCs from rubber infill can be aerosolized into respirable form during sports play. The authors calculated health risk assuming the use of recycled rubber granulate, which releases the lowest amounts of these chemicals of any type of rubber infill.

The report concluded that, given current knowledge, the use of synthetic turf indoors does not cause any elevated health risk, even in vulnerable populations such as children. However, the report continues, “It should also be noted that little or no toxicological information is available for many of the volatile organic compounds which have been demonstrated as being present in the air in the [indoor stadiums]. . . . [Furthermore], not all organic compounds in the [stadium] air have been identified.” In particular the report called for more information regarding the development of asthma and airway allergies in response to exposure to the latex in many tires.

Similarly, the California Office of Environmental Health Hazard Assessment (OEHHA), in the January 2007 report *Evaluation of Health Effects of Recycled Waste Tires in Playground and Track Products*, concluded that 49 chemicals could be released from tire crumbs. Based on an experiment simulating gastric digestion, the OEHHA calculated a cancer risk of 1.2 in 10 million assuming a one-time ingestion over a lifetime—well below the 1 in 1 million *di minimis* risk threshold. In a hand-wipe experiment, the OEHHA calculated an increased cancer risk of 2.9 in 1 million for ingestion of chrysene (a suspected human carcinogen found in tire rubber) via hand-to-mouth contact with crumb rubber infill. This estimate assumed regular playground use for the first 12 years of life and was termed by the authors to be “slightly higher” than the *di minimis* level.

In the summer of 2007, Environment and Human Health, Inc. (EHHI), a nonprofit organization headquartered in North Haven, Connecticut, commissioned a study from the Connecticut Agricultural Experiment Station to determine whether toxic compounds from crumb rubber could be released into air or water. The report *Artificial Turf* describes identifying 25 chemical species with 72–99% certainty using mass spectrometry–gas chromatography. Among those definitively confirmed were the irritants benzothiazole and *n*-hexadecane; butylated hydroxyanisole, a carcinogen and suspected endocrine disruptor; and 4-(*t*-octyl) phenol, a corrosive that can be injurious to mucous membranes.

The Synthetic Turf Council said in a statement issued on 13 December 2007 that “Claims of toxicity [in the EHHI report] are based on extreme laboratory testing such as the use of solvents and high temperatures to generate pollutants.” But the EHHI stands by its studies. *Artifical Turf* author David Brown, EHHI’s director of public health toxicology, says, “It is clear the recycled rubber crumbs are not inert, nor is a high temperature or severe solvent extraction needed to release metals, volatile, or semi-volatile organic compounds.” Brown asserts that the laboratory tests approximate conditions that can be found on the field, and that no solvent besides water was used.

According to Brown, the basic barrier to accurately assessing the safety of recycled tire rubber is the high variability in tire construction and the lack of chemical characterization of the crumb rubber. “Very few samples have been tested,” he says. “There is no study with sufficient sample sizes to determine the potential hazard.” He adds, “Since new tires contain vastly different amounts of the toxic materials, based on the intended use, it is impossible to ensure players or gardeners and others that their personal exposure is within safe limits.”

Another debated health issue is that of injuries. Several studies published in a supplement to the August 2007 issue of the *British Journal of Sports Medicine* reported no differences in the incidence, severity, nature, or cause of injuries in soccer teams who played on grass versus new-generation synthetic turf. However, injuries may depend on the type of sport being played. A five-year prospective study of football injuries among high school teams published 1 October 2004 in *The American Journal of Sports Medicine* showed that there were about 10% more injuries when games were played on synthetic turf than when played on grass surfaces. Conversely, the risk of serious head and knee injuries was greater on grass fields.

Injuries lead to another concern: infection with methicillin-resistant *Staphylococcus aureus* (MRSA), which is thought to spread especially easily among athletes because of repeated skin-to-skin contact, frequency of cuts and abrasions, and sharing of locker room space and equipment. A study conducted by the Centers for Disease Control and Prevention and published in the 3 February 2005 issue of the *New England Journal of Medicine* showed that, although synthetic turf itself did not appear to harbor MRSA, the greater number of turf burns caused by the abrasive friction of this type of surface increased the probability of MRSA infection, especially among professional athletes playing on hard surfaces.

There is, however, some evidence to suggest that synthetic turf may harbor more bacteria. For example, an industry study sponsored by Sprinturf, a maker of synthetic turf, found that infill containing a sand/rubber mixture had 50,000 times higher levels of bacteria than infill made of rubber alone. To address this, the company markets synthetic turf that is “sand-free” as a safer alternative and offers sanitation for those fields already installed.

Proper maintenance of synthetic turf requires that the fields be sanitized to remove bodily fluids and animal droppings; manufacturers market sanitizing products for this purpose. According to *Synthetic Turf Sports Fields: A Construction and Maintenance Manual*, published in 2006 by the American Sports Builders Association, some synthetic turf owners disinfect their fields as often as twice a month, with more frequent cleanings for sideline areas, where contaminants concentrate.

## Different Shades of Green

Cultivated natural grass carries plenty of environmental baggage. According to “Water Management on Turfgrass,” a paper on the Texas A&M University Cooperative Extension website (http://plantanswers.tamu.edu/), natural grass sports fields can require up to 1.5 million gallons of water per acre per year. The f r e q u e n t m o w i n g required for natural grass lawns and fields also results in emissions of hydrocarbons and carbon monoxide (up to 5% of such emissions in the United States, according to the Environmental Protection Agency).

Natural grass does offer tangible benefits, however. According to Turfgrass Producers International, these include increased pollution control, absorption of carbon dioxide, a cooling effect, water filtration, and prevention of soil erosion. There are also perhaps intangible benefits to a field of grass. Crain presents the idea that replacing grass with synthetic turf can hinder children’s creative play and affect their development. “Today’s children largely grow up in synthetic, indoor environments,” he says. “Now, with the growing popularity of synthetic turf fields, their experience with nature will be less than ever.”

Adds Croft, “Although there is an important need for open spaces, the issue here is not open space but active recreational facilities. I don’t see the connection between open space and installing synthetic turf fields.”

Synthetic turf does offer certain advantages over natural grass. *A New Turf War: Synthetic Turf in New York City Parks*, a report released in 2006 by the advocacy group New Yorkers for Parks, points out, “Proponents of synthetic turf fields tout the reduction of allergy and asthma triggers. The removal of natural pollens and grasses may be beneficial to children and adults with these afflictions.”

One of the main arguments used in favor of synthetic turf is that it can be installed relatively quickly and, once functional, can be used almost continuously. In contrast, grass fields need time to take root and must be closed periodically for proper maintenance. For example, the Central Park Conservancy, a private philanthropy that maintains New York City’s Central Park, closes grass fields all winter; during the summer and spring, fields are closed on a rotating basis for restoration. Also, tackle football and cleated shoes are prohibited on all of the fields, and the fields are closed whenever it rains or they are wet. According to estimates from the New York City Department of Parks & Recreation, synthetic fields can be open for use 28% more of the time in a year than natural grass fields because they can withstand heavy use, which the department estimates has doubled in the last eight years.

Lower cost for long-term maintenance is another argument that is made for synthetic turf, although the degree of the savings is disputed. It is generally agreed that installation costs of synthetic turf can be almost double those of natural grass. For instance, a synthetic turf soccer field can cost almost $1.4 million compared with a natural grass field at about $690,000. But when the costs are prorated over the expected lifespan of the field, including maintenance, the difference in cost narrows to less than $15,000 more for the natural grass, according to *A New Turf War*.

Although some, like Benepe, consider this cost savings to be substantial, others consider it insignificant. As Christian DiPalermo, executive director of New Yorkers for Parks, puts it, “The amount of money saved is negligible considering the many unknowns about artificial turf.”

One drawback that both fans and critics of synthetic turf agree on is that these fields can get much hotter than natural grass. Stuart Gaffin, an associate research scientist at the Center for Climate Systems Research at Columbia University, initially became involved with the temperature issues of synthetic turf fields while conducting studies for another project on the cooling benefits of urban trees and parks. Using thermal satellite images and geographic information systems, Gaffin noticed that a number of the hottest spots in the city turned out to be synthetic turf fields.

Direct temperature measurements conducted during site visits showed that synthetic turf fields can get up to 60° hotter than grass, with surface temperatures reaching 160°F on summer days. For example, on 6 July 2007, a day in which the atmospheric temperature was 78°F in the early afternoon, the temperature on a grass field that was receiving direct sunlight was 85°F while an adjacent synthetic turf field had heated to 140°F. “Exposures of ten minutes or longer to surface temperatures above 122°F can cause skin injuries, so this is a real concern,” said Joel Forman, medical director of the Pediatric Environmental Health Specialty Unit at Mount Sinai School of Medicine, speaking at a 6 December 2007 symposium on the issue.

Many physical properties of synthetic turf—including its dark pigments, low-density mass, and lack of ability to vaporize water and cool the surrounding air—make it particularly efficient at increasing its temperature when exposed to the sun. This is not only a hazard for users, but also can contribute to the “heat island effect,” in which cities become hotter than surrounding areas because of heat absorbed by dark man-made surfaces such as roofs and asphalt. From many site visits to both black roofs and synthetic turf fields, Gaffin has concluded that the fields rival black roofs in their elevated surface temperatures.

Although it is often argued that one of the advantages of synthetic turf is that it does not need irrigation, some installations must be watered to control the excessive heat. Benepe stated in public hearings that water misters may have to be installed in some fields to help remedy the heat problem. According to Gaffin, synthetic turf is so efficient at absorbing sunlight, that cooling with water is only temporarily effective. “After a short while of watering, I expect the temperature should rebound and the surface become intolerably hot again,” he says.

In addition to heat control, the International Hockey Federation requires that college teams saturate synthetic turf fields before each practice and game to increase traction, according to an article in the 19 October 2007 Raleigh (North Carolina) *News & Observer*. The article, which examined why local universities were watering their synthetic turf fields in the midst of severe ongoing drought in the U.S. Southeast, noted that Duke University received a business exemption to water the fields provided overall campus water consumption decreased by 30%.

The EHHI study addressed the question of whether synthetic turf fields can contribute to increased water contamination from rain or from spraying or misting. The study found that 25 different chemical species and 4 metals (zinc, selenium, lead, and cadmium) could be released into water from rubber infill. Moreover, because synthetic turf is unable to absorb or filter rain-water, chemicals filter directly into storm drains and into the municipal sewer system without the beneficial filtration that live vegetation provides. Benepe and others agree this can be an issue that New York City would need to address, as water runoff from synthetic turf fields could overwhelm storm drains, thus contributing to the estimated 27 billion gallons of raw sewage and stormwater that discharge from 460 combined sewer overflows into New York Harbor each year.

Finally, what happens to synthetic turf fields when they are no longer usable? Industry estimates that synthetic turf fields have a lifespan of 10 to 12 years, whereupon the material must be disposed of appropriately. Rick Doyle, president of the Synthetic Turf Council, says the infill could be cleaned and reused; put to another purpose, such as for rubber asphalt; incinerated; used in place of soil to separate landfill layers; or otherwise recycled. Typically, however, it is landfilled.

## Alternatives

One of the benefits of synthetic turf is that it can serve as a way to reuse old tires, a real problem given the 1 billion–plus tires that are sold every year. Doyle says the synthetic turf industry currently recycles one-twelfth of the 300 million auto tires that are withdrawn from use each year. The average soccer field can contain crumb rubber made from 27,000 tires at a density of about 4 to 15 pounds of infill per square foot.

Europe has launched an aggressive tire recovery campaign in which tires that meet quality criteria can be retreaded and reused. End-of-life tires that cannot be reused are recycled for other uses including some industrial energy-generating applications, the production of rubberized pavement, and recycling into materials for the car industry (in addition to some use in producing synthetic turf). In western Europe, recovery rates of used tires have increased from 65% in 2001 to almost 90% in 2005.

Whereas end-of-life tires add tons of waste a year for disposal in many areas, in Europe they are turning into a potentially lucrative secondary raw material. “There are increasingly numerous applications,” says Serge Palard, head of the end-of-life tire recovery department at Michelin, one of the largest tire manufacturers in the world. “In some countries where we did not know what to do with end-of-life tires a few years ago, now we do not have enough to meet the demand of all the reprocessors.”

In accordance with the European Union’s recently implemented REACH (Registration, Evaluation, Authorisation and Restriction of Chemicals) regulations, which will require more testing of industrial chemicals, companies such as Michelin are working to reduce the use of harmful chemicals in tires in order to facilitate recycling into other products.

European companies are also finding innovative ways to address concerns regarding recycled tire infill in synthetic turf. In Italy, for example, there is an effort to market synthetic turf fields that feature infill made of a new thermoplastic material that is thought to be nontoxic. Mondo, a manufacturer of floor surfaces, produces Ecofill, a patented polyolefin-based granule used in synthetic turf. According to the company, this material disperses heat more efficiently; is highly shock absorbent; does not contain polyvinyl chloride, chlorine, plasticizers, heavy metals, or other harmful chemicals; and is 100% recyclable.

Another alternative is infill made from plant-derived materials. Synthetic turf manufacturer Limonta Sport produces Geo Safe Play, an infill made from coconut husks and cork. Company spokesperson Domenic Carapella says, “There are certainly alternatives to crumb rubber. There is no longer a reason to sacrifice the playing quality and more importantly the health of children [playing on synthetic turf].”

Why can’t the alternative to bad grass fields simply be well-maintained grass fields, asks Croft. Certain varieties of turf grasses have been bred for resistance to stress, ability to withstand trampling and low water conditions, and other characteristics that make them appropriate for athletic field use.

But according to Doyle, increased maintenance is not the answer. “More maintenance cannot overcome overusage of a natural grass sports field,” he says. “And overusage of a natural grass sports field or usage during a rainstorm or in months of dormancy will produce an unsafe playing surface.” Adds Benepe, “Even the wealthiest professional sports teams and Ivy League universities have concluded that grass fields are a losing proposition for intense-use sports such as football or soccer. . . .There is also the reality that natural turf fields used for high-intensity sports must be replaced every few years, unless you severely restrict use.”

For now, New York State Assembly-members Steve Englebright, William Colton, and David Koon have proposed legislation to impose a six-month moratorium on the installation of synthetic turf until the state health and conservation departments have better studied the pros and cons of natural and synthetic grass. Said Englebright in a 5 November 2007 statement, “Before we take risks with our children’s health and drinking water quality, we need to make sure that the uncertainties . . . are fully investigated.”

## Figures and Tables

**Figure:**
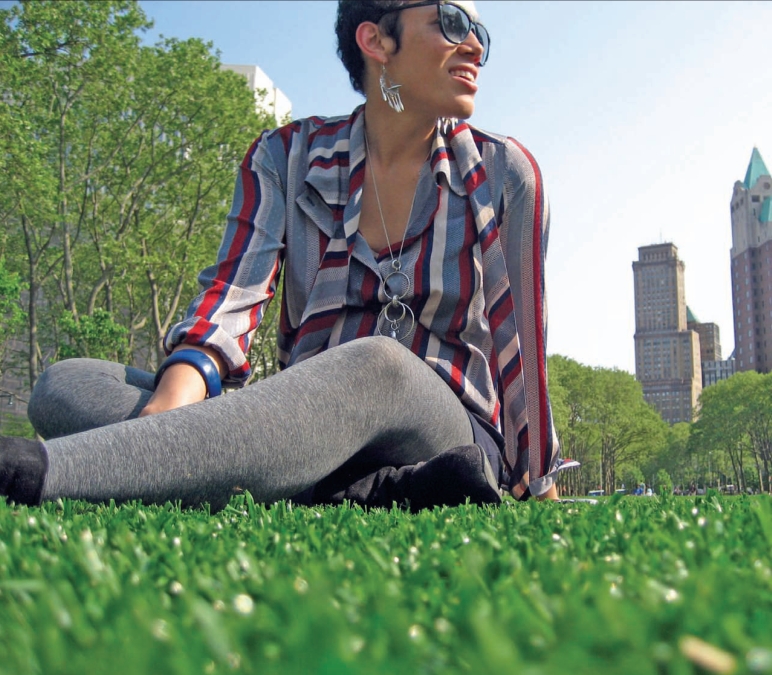
Synthetic turf field at Cadman Plaza Park, Brooklyn, New York

**Figure:**
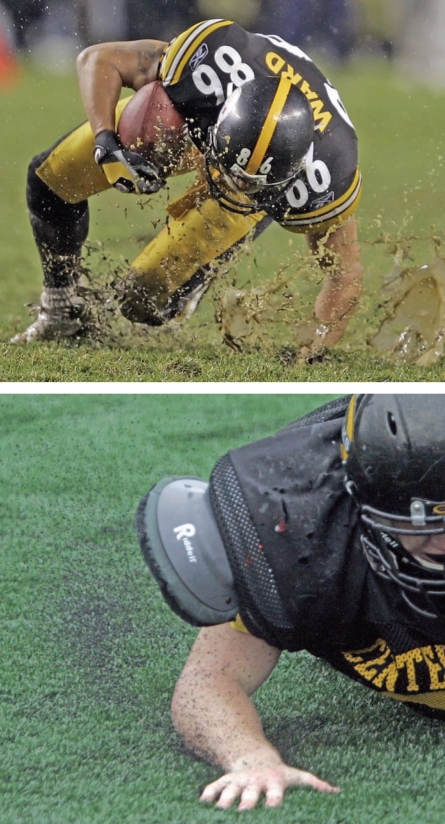
Weighing costs and benefits High-impact sports take a toll on natural grass fields (above), but concerns about the unknown health effects of crumb rubber infill (seen below spraying upon impact) and other environmental health concerns keep some stakeholders from wholeheartedly buying in to the benefits of synthetic turf.

**Figure:**
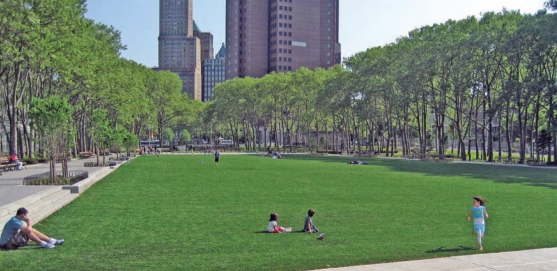
Moratorium introduced Cadman Plaza Park in Brooklyn, New York, boasts a picture-perfect blanket of synthetic turf. In fall 2007 the New York State Assembly has introduced a moratorium on the sale and installation of synthetic turf containing crumb rubber until the state completes a comprehensive study on its potential health effects.

**Figure:**
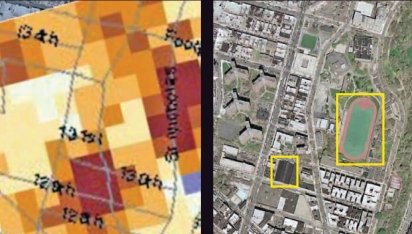
Thermal effect An image taken 14 August 2002 by NASA’s Landsat satellite (left) shows surface temperatures in upper Manhattan (red indicates warm temperatures, and blue indicates cool temperatures). A large synthetic turf field created high temperatures similar to those on a large black roof (see Google Earth image, right). Cool spots almost always correspond to urban vegetation, such as parks, street trees, and water bodies.

**Figure:**
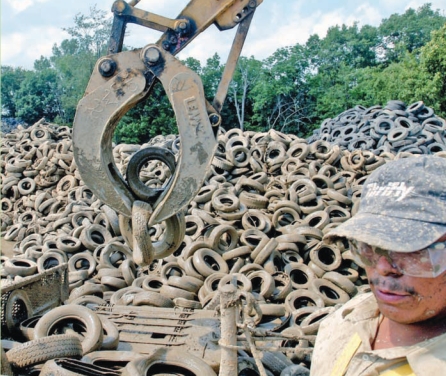
Growing demand Expanding markets for the reuse of scrap tires have enabled the state of Ohio (home to this recycling site) to reduce its stockpiles faster than anticipated. In Europe, some countries are having trouble meeting the demand for end-of-life tires to recycle.

